# Attitudes of psychotherapists towards their own performance and the role of the social comparison group: The self-assessment bias in psychodynamic, humanistic, systemic, and behavioral therapists

**DOI:** 10.3389/fpsyg.2022.966947

**Published:** 2022-10-13

**Authors:** Thomas Probst, Elke Humer, Andrea Jesser, Christoph Pieh

**Affiliations:** Department for Psychosomatic Medicine and Psychotherapy, University for Continuing Education Krems, Krems, Austria

**Keywords:** psychotherapy, self-assessment, self-assessment bias, performance, therapeutic orientations

## Abstract

Studies report that psychotherapists overestimate their own performance (self-assessment bias). This study aimed to examine if the self-assessment bias in psychotherapists differs between therapeutic orientations and/or between social comparison groups. Psychotherapists gave subjective estimations of their professional performance (0–100 scale from poorest to best performance) compared to two social comparison groups (“all psychotherapists” vs. “psychotherapists with the same therapeutic approach”). They further rated the proportion of their patients recovering, improving, not changing, or deteriorating. In total, *N* = 229 Austrian psychotherapists (*n* = 39 psychodynamic, *n* = 121 humanistic, *n* = 48 systemic, *n* = 21 behavioral) participated in the online survey. Psychotherapists rated their own performance on average at *M* = 79.11 relative to “all psychotherapists” vs. at *M* = 77.76 relative to “psychotherapists with the same therapeutic approach” (*p* < 0.05). This was not significantly different between therapeutic orientations. A significant interaction between social comparison group and therapeutic orientation (*p* < 0.05) revealed a drop of self-assessement bias in social comparison group “same approach” vs. “all psychotherapists” in psychodynamic and humanistic therapists (*p* < 0.05). Psychotherapists overestimated the proportion of patients recovering (*M* = 44.76%), improving (*M* = 43.73%) and underestimated the proportion of patients not changing (*M* = 9.86%) and deteriorating (*M* = 1.64%), with no differences between orientations. The self-assessment bias did not differ between therapeutic orientations, but the social comparison group appears to be an important variable. A major drawback is that results have not been connected to patient-reported outcome or objectively rated performance parameters.

## Introduction

Research has shown that self-reported measures of therapeutic skills and patient’s progress are systematically overestimated by psychotherapists (Macdonald and Mellor-Clark, 2015). Self-assessment bias means overestimation of one’s own professional performance relative to a social comparison group (SCG) (Walfish et al., 2012). Research on the relationship between self-perception and social perception has shown the generality of a self–other asymmetry (Pronin et al., 2004). This asymmetry arises from the observation that other people’s responses to a situation sometimes differ from our own. When trying to make sense of this difference in response, we try to draw on attributions about the underlying characteristics of the other and the other’s views and priorities. In general, we assume that our perspective on a given situation is objective and thus superior to others. Consequently, we are more ready to detect or infer a bias in the other. In essence, this corresponds to the epistemological stance of naive realism (Ross and Ward, 1996). About the self-assessment bias, this means that people are more ready to view their assessments and outcomes in a positive light than those of others. This has been shown in a variety of areas of performance (Dunning et al., 2004), including health care professionals. In the United States, a mean rating of mental health care professionals’ own performance relative to SCG of others with similar qualifications at the 80th centile was found (Walfish et al., 2012), whereas the 50th could be expected due to variability between therapists (Okiishi et al., 2003). For United Kingdom mental health care providers (Parker and Waller, 2015), a smaller self-assessment bias than in the United States study (Walfish et al., 2012) relative to SCG of others with similar qualifications was observed. Both studies asked about estimations of one’s own performance relative to one SCG “others with similar qualifications.” Festingers’ social comparison theory (Festinger, 1954), however, highlights the importance of the social comparison group (degree of closeness/divergence). Also, a study on pupils observed variations in self-assessment with the specific definition of the SCG (Blatchford, 1997). Academic self-assessment dropped from the SCG “all children of the same age” to the SCG “same class” (Blatchford, 1997). Whether self-assessment of psychotherapists changes with the SCG (“all psychotherapists” vs. “psychotherapists with the same psychotherapeutic approach”) has not been assessed so far.

Although overly positive self-views might have also favorable consequences under certain circumstances (Walfish et al., 2012), systematically overestimating skills, expertise and knowledge not only prevents therapists from professional development but also increases the risk of not recognizing that some of their patients do not benefit from psychotherapy or even begin to deteriorate (Walfish et al., 2012; Macdonald and Mellor-Clark, 2015). Mental health care professionals` perceptions of patient progress might even be more important than estimations of their abilities (Walfish et al., 2012). As even among the most effective therapists a proportion of patients deteriorate (Okiishi et al., 2006), recognizing that patients are at risk of treatment failure is essential to improve patient outcomes and decrease the proportion of patients that deteriorate or drop out (Lambert et al., 2002; Hawkins et al., 2004). Studies in the United States and United Kingdom observed that therapists overestimate recovering/improving patients and underestimate deteriorating patients compared to rates found in the outcome literature (Walfish et al., 2012; Parker and Waller, 2015). The inability to recognize patients who worsen during psychotherapy has even been observed in psychotherapists who were informed about a deterioration rate of 8% (Hannan et al., 2005).

It has been reported that in routine practice a considerable proportion of patients does not recover and some even experience reliable deterioration (Hansen et al., 2002; Westbrook and Kirk, 2005). Inaccurate self-assessments might be one potential reason, as psychotherapists who estimate their own skills above average, are likely less willing to monitor their patient’s outcomes and to take measures to improve their skills and outcomes (Walfish et al., 2012; Parker and Waller, 2015). Thus, it is essential to determine potentials flaws in self-assessment in psychotherapists to provide implications for the practice of psychotherapy on the necessity to implement tools to repair potential self-assessment biases (Walfish et al., 2012). Feedback-informed treatment has been suggested as helpful for psychotherapists to become aware of patients who deteriorate during treatment and thus could serve as a countermeasure for self-assessment bias (Hannan et al., 2005). Whether self-assessment bias differs among therapeutic orientations and thus whether feedback-informed treatment should be prioritized in therapeutic orientations that are specifically prone toward self-assessment bias has not been studied so far. The current study aimed to examine the self-assessment bias in a sample of Austrian psychotherapists. Austria has a wide range of established psychotherapy approaches, which can be classified into four orientations (psychodynamic, humanistic, systemic, and behavioral) (Heidegger, 2017). The main difference between the orientations resides in their understanding of the human psyche and their theories regarding the origin of mental disorders (DiTomasso et al., 2009; Pocock, 2015; Schlippe Von and Schweitzer, 2015; Cabaniss, 2016). Accordingly, different interventions are used in the treatment, and to some extent, the setting differs, e.g., the frequency of sessions and the duration of psychotherapy. Against the background of the differences as well as similarities between the four orientations, this study aimed to investigate if the self-assessment bias in psychotherapists differs between the four therapeutic orientations and/or between social comparison groups (i.e., estimating their own performance relative to “all other psychotherapists” vs. “psychotherapists with the same therapeutic approach”). We hypothesized that irrespective of the therapeutic orientation psychotherapists overestimate their own professional performance and patient progress. Based on the human tendency to assume that people who have a similar view of the world will handle a situation similar to what we do and that people whose views differ from our own are more likely to be biased, it seems likely that differences will emerge when comparing SCGs. Thus, we further hypothesized that the self-assessment bias is reduced in a social comparison group of more closeness.

## Materials and methods

### Study design

The present study is based on an online survey with *N* = 238 Austrian psychotherapists, conducted between February and April 2021 [more details in (Humer et al., 2021)]. Psychotherapists received the link to the anonymized online survey by e-mails from the study team and the Austrian Federal Association for Psychotherapy (Vienna, Austria) supported recruitment for this study by sending their members an information e-mail. The study was conducted following the American Association for Public Opinion Research (AAPOR) reporting guidelines (Pitt et al., 2021) and according to the guidelines of the Declaration of Helsinki and approved by the Ethics Committee of the Danube University Krems (protocol code EKGZ23-2018-2021).

In Austria, psychotherapy is a profession regulated by law. Psychotherapy training is offered by institutions accredited by the Austrian Federal Ministry of Social Affairs, Health, Care, and Consumer Protection. Training is regulated by the Austrian psychotherapy law and comprises two parts: first 765 h of lessons in theory and 550 h of practical training, second 300 advanced lessons in theory and 1,600 h of practical training. In Austria, a total of 23 psychotherapeutic approaches are accredited (Heidegger, 2017), which can be categorized into four psychotherapeutic orientations (psychodynamic, humanistic, systemic, and behavioral).

### Measures

The online survey included information on gender, age, years in the profession, and therapeutic approach. Self-assessment of their professional performance as well as their estimates on therapeutic outcomes were assessed with the two measures applied in the aforementioned studies (Walfish et al., 2012; Parker and Waller, 2015). First, psychotherapists were asked to respond to the following two questions:

1. How would you rate the quality of your psychotherapeutic sessions on average, in comparison to all other psychotherapists in Austria of all 23 psychotherapeutic approaches who have similar qualifications/experience as you?
2. How would you rate the quality of your psychotherapeutic sessions on average, in comparison to all other psychotherapists in Austria who are trained in the same psychotherapeutic approach as you are and have similar qualifications/experience as you?

Psychotherapists were asked to rate both questions on a 0–100 scale, with 0 = poorest, 50 = average, and 100 = best. Extending the research conducted by Walfish et al. (2012) and Parker and Waller (2015), who asked about estimations of one’s performance relative to solely one SCG (“others with similar qualification”), two different SCGs were investigated in the study at hand. The reason for including two different SCGs is based on previous studies highlighting the importance of the social comparison group (degree of closeness/divergence) in self-assessments (Festinger, 1954; Blatchford, 1997). Second, psychotherapists were asked to rate (0–100% scales) the proportion of their patients recovering, improving, not changing, or deteriorating. The following definitions were provided: recovering: no symptoms at the end of treatment and no need for further psychotherapeutic treatment; improving: significant reduction of symptoms at end of treatment, but still some problems; not changing: no change at end of treatment; deteriorating: significant increase in symptoms at the end of treatment.

### Statistics

To analyze the first measure, a repeated measure ANOVA was applied (between-subject effect “orientation” 4-levels, within-subject effect “SCG” 2-levels “all” vs. “same”). To analyze the second measure (% of recovered, improved, unchanged, and deteriorated patients), univariate ANOVAs (between-subject factor “orientation” 4-levels) were used.

## Results

Of the total *N* = 238 participating psychotherapists (Humer et al., 2021), nine psychotherapists were excluded from further analyses as they could not be assigned to one of the four orientations. The sample characteristics of the remaining *N* = 229 psychotherapists are presented in Table 1. More than half of the psychotherapists were trained in humanistic approaches (*n* = 121), while behavioral psychotherapists represented the smallest group (*n* = 21). More than three-quarters were female. Their mean age was 50.66 (*SD* = 9.90) years and they were on average 12.70 (*SD* = 9.39) years in the profession. There were no significant differences between the therapeutic orientations in gender, age, and professional experience. Results of both measures of the self-assessment bias are summarized in Table 2. All measures of self-assessment bias were not affected by the therapeutic orientation (main effect “orientation” > 0.05). No one rated their own performance below the average of 50. The main effect for “SCG” [*F*(1,225 = 4.888; *p* = 0.028] in the first measure showed that, in general, self-assessment bias was smaller for the same approach vs. all psychotherapists. Bonferroni-corrected post-hoc tests to explore the interaction “orientation x SCG” [*F*(3,225 = 4.427, *p* = 0.005] revealed that psychodynamic (*p* = 0.004) and humanistic psychotherapists (*p* = 0.035) had a drop of self-assessment bias from SCG “all approaches” to “same approach.” Psychotherapists estimated that on average 44.76% of their patients recover and 43.73% improve. The proportion of patients not changing was estimated to amount to 9.86%. A mean proportion of 1.64% of the treated patients was estimated to deteriorate.

**Table 1:** Sample characteristics and comparison between the four therapeutic orientations in gender, age, and professional years.

Variable	All (*N* = 229)	Psychodynamic (*n* = 39)	Humanistic (*n* = 121)	Systemic (*n* = 48)	Behavioral (*n* = 21)	Statistics
Gender (% female)	76.42	79.49	71.90	81.25	85.71	χ^2^(6) = 8.478; *p* = 0.205
Age [*M* (*SD)* in years]	50.66 (9.90)	49.28 (10.39)	51.92 (9.35)	50.71 (9.69)	45.86 (11.34)	*F*(3,225) = 2.606; *p* = 0.053
Professional experience [*M* (*SD)* in years]	12.70 (9.39)	10.23 (7.86)	13.46 (9.95)	12.21 (9.20)	14.06 (8.72)	*F*(3,208) = 1.219; *p* = 0.304

Nine therapists trained in more than one therapeutic orientation were excluded. Professional years was available for *N* = 212 psychotherapists (*n* = 35 psychodynamic, *n* = 113 humanistic, *n* = 47 systemic, *n* = 17 behavioral).

**Table 2:** Results for the two measures of the self-assessment bias.

Variable		All (*N* = 229)	Psycho-dynamic (*n* = 39)	Humanistic (*n* = 121)	Systemic (*n* = 48)	Behavioral (*n* = 21)	Statistics
Measure 1 to assess self-assessment^1^: Estimation of one’s own performance (0–100 scale: 0 = poorest, 50 = average, 100 = best)…	… relative to SCG all psychotherapists *M* (*SD*) [min/max]	79.11 (14.41) [50/100]	77.54 (16.35) [50/100]	79.07 (13.54) [50/100]	78.92 (15.47) [50/100]	82.67 (13.29) [50/100]	ME orientation: *F*(3,225) = 1.063; *p* = 0.366; η_p_^2^ = 0.014 ME SCG: *F*(1,225) = 4.888; *p* = 0.028; η_p_^2^ = 0.021 IE orientation x SCG: *F*(3,225) = 4.427; *p* = 0.005 η_p_^2^ = 0.056
	… relative to SCG psychotherapists with the same therapeutic approach *M* (*SD*) [min/max]	77.76 (15.54) [50/100]	73.41 (16.89) [50/100]	77.38 (14.85) [50/100]	81.29 (16.28) [50/100]	79.90 (13.93) [50/96]	
**Variable**		**All (*N* = 225)**	**Psycho-dynamic (*n* = 39)**	**Humanistic (*n* = 118)**	**Systemic (*n* = 48)**	**Behavioral (*n* = 20)**	**Statistics**
Measure 2 to assess self-assessment^2^: The proportion of own patients recovering, improving, not changing, deteriorating (0–100% scale each)	% recovering *M* (*SD*) [min/max]	44.76 (22.65) [0/100]	41.46 (25.00) [0/80]	44.14 (22.27) [5/100]	49.81 (21.69) [10/90]	42.75 (22.03) [5/75]	ME orientation: *F*(3,221) = 1.157; *p* = 0.327; η_p_^2^ = 0.015
	% improving *M* (*SD*) [min/max]	43.73 (21.23) [0/100]	46.74 (23.00) [15/100]	44.94 (21.19) [0/90]	38.31 (19.35) [9/87]	43.75 (21.51) [15/80]	ME orientation: *F*(3,221) = 1.440; *p* = 0.232; η_p_^2^ = 0.019
	% not changing *M* (*SD*) [min/max]	9.86 (7.38) [0/35]	9.51 (8.40) [0/35]	9.69 (7.24) [0/35]	10.00 (7.45) [0/35]	11.20 (6.25) [0/20]	ME orientation: *F*(3,221) = 0.271; *p* = 0.846; η_p_^2^=0.004
	% deteriorating *M* (*SD*) [min/max]	1.64 (3.42) [0/25]	2.28 (4.61) [0/25]	1.23 (2.19) [0/10]	1.88 (4.13) [0/20]	2.30 (4.61) [0/20]	ME orientation: *F*(3,221) = 1.357; *p* = 0.257; η_p_^2^ = 0.018

SCG, social comparison group; ME, main effect; IE, interaction effect; η_p_^2^, partial eta-squared.

1 Nine therapists trained in more than one therapeutic orientation were excluded.

2 Nine therapists trained in more than one therapeutic orientation and four therapists with the sum of answers % recovered + % improved + % not changed + % deteriorate not adding up to 100% were excluded.

## Discussion

Results suggest that irrespective of the therapeutic orientation, psychotherapists rate their own professional performance above average when compared to colleagues. This overestimation is more pronounced when they compare their performance with all other psychotherapists as compared to those with the same psychotherapeutic orientation. Moreover, data suggest that psychotherapists overestimate their effectiveness in terms of overestimating positive patient outcomes and underrecognizing patients that deteriorate during treatment.

Compared to other countries, evidence-based practice is still not very popular within the psychotherapy community in Austria (Nussbaumer-Streit et al., 2022). Not all of the 23 psychotherapeutic approaches accredited in Austria fulfill the criteria to be evidence-based. Also, academic education is not a prerequisite for the psychotherapeutic profession in Austria (Heidegger, 2017). Compared to other countries, the belief that overall competence is more important than science and that psychotherapy is more of an art than a science is more common among Austrian psychotherapists (Nussbaumer-Streit et al., 2022). Despite the less favorable attitude toward evidence-based practice in Austrian psychotherapists, the extent of the self-assessment bias found in the present study corresponds to findings of previous studies, especially those from the United States (Walfish et al., 2012; Parker and Waller, 2015). Karpen (2018) argued that the psychological mechanisms underlying biased self-assessment occur on an unconscious level. He concluded that strategies aiming at addressing the bias directly, such as informing people about typical biases, aim at the wrong direction. Instead, he opted for strategies that prevent the unconscious biasing mechanisms from operating. For instance, video-informed feedback from educators and colleagues as well as guidance on improvement strategies are effective in reducing self-assessment bias (Lane and Gottlieb, 2004).

In line with our hypothesis and the results by Blatchford (1997), self-assessment bias was reduced in an SCG of more closeness. Psychodynamic and humanistic therapists in particular assessed their performance closer to the performance of therapists of the same orientation than in comparison to all other therapists. This might be related to different reasons. First, differences might be related to methodological issues. Due to the differences in sample size between the four orientations, the significant effect observed in humanistic psychotherapists for the differences between self-assessment relative to the same approach vs. all psychotherapists should be interpreted with caution. As larger sample sizes enable statistical tests to detect smaller differences between groups, the significance is likely attributable to the relatively big sample size (*n* = 121). Absolute differences between self-assessment relative to the same approach vs. all psychotherapists were even numerically lower as compared to systemic or behavioral therapists. Second, psychotherapists likely know psychotherapists from one’s own orientation better than colleagues from other orientations, which likely led to a more realistic comparison with their skills. This finding is in line with results observed in students, showing a drop in self-assessment bias in a SCG of more closeness (Blatchford, 1997). Third, ingroup-outgroup dynamics might also play a role. Ingroup members see themselves as more similar and rate themselves as less different than outgroup members (Mussweiler and Bodenhausen, 2002).

Results also confirmed our hypothesis that irrespective of the therapeutic orientation psychotherapists overestimate their patient’s progress. On average psychotherapists estimated that an average of 1.64% of their patients leave treatment worse off than they began treatment. In contrast, it has been estimated that in 5 to 10% of adult patients participating in clinical trials, therapies have negative than rather positive consequences for the patients (Lambert and Ogles, 2004). In routine care and youths, the situation seems to be even more problematic, with deterioration rates as high as 24% (Warren et al., 2010). Results of the present study further highlight, that psychotherapists often overestimate their own patient’s progress and overlook worsening states. Monitoring patient progress and using outside feedback provides an integral part of evidence-based practice in mental health and might help psychotherapists to become aware of patients who deteriorate during treatment (Jensen-Doss et al., 2018). A growing body of evidence supports the notion that feedback-informed treatment improves performance of psychotehrapists and treatment outcomes (Miller et al., 2016).

As overestimation of professional performance and patient outcomes were observed in all therapeutic orientations to a similar extent, the results of the present study imply that it is not necessary to prioritize feedback-informed treatment implementation in specific therapeutic orientations.

Future research should compare the therapists’ self-assessments of their performance with their actual patient-reported outcomes. In addition, more social comparison groups could be investigated (e. g., gender, age, training institution, etc). Limitations of this study are that no pre-post or follow-up patient-reported outcomes were assessed and the missing objectively rated performance parameters. Therefore it is also possible that a potential self-selection bias toward more skilled psychotherapists led to an accurate reflection of their performance levels rather than a self-assemsent bias. A further drawback is the relatively small sample size, especially in the subgroups.

## Data availability statement

The raw data supporting the conclusions of this article will be made available by the authors, without undue reservation.

## Ethics statement

The studies involving human participants were reviewed and approved by Ethics Committee of the Danube University Krems, Austria. The patients/participants provided their written informed consent to participate in this study.

## Author contributions

TP recruited the participants, designed the study, performed the statitical analyses, and wrote the manuscript. EH, AJ, and CP co-designed the study and reviewed the manuscript. All authors contributed to the article and approved the submitted version.

## Conflict of interest

The authors declare that the research was conducted in the absence of any commercial or financial relationships that could be construed as a potential conflict of interest.

## Publisher’s note

All claims expressed in this article are solely those of the authors and do not necessarily represent those of their affiliated organizations, or those of the publisher, the editors and the reviewers. Any product that may be evaluated in this article, or claim that may be made by its manufacturer, is not guaranteed or endorsed by the publisher.
